# Coordination of glucose monitoring, self-care behaviour and mental health: achieving precision monitoring in diabetes

**DOI:** 10.1007/s00125-022-05685-7

**Published:** 2022-04-05

**Authors:** Norbert Hermanns, Dominic Ehrmann, Amit Shapira, Bernhard Kulzer, Andreas Schmitt, Lori Laffel

**Affiliations:** 1 Research Institute Diabetes Academy Mergentheim (FIDAM), Bad Mergentheim, Germany; 2 Department of Clinical Psychology and Psychotherapy, University of Bamberg, Bamberg, Germany; 3 German Center for Diabetes Research (DZD), Muenchen-Neuherberg, Germany; 4 Harvard Medical School, Joslin Diabetes Center, Boston, MA USA; 5 Harvard Medical School, Boston Children’s Hospital, Boston, MA USA

**Keywords:** Behavioural parameters, Diabetes, Ecological momentary assessment, Glucose monitoring, Mental health, Personalised medicine, Precision medicine, Review

## Abstract

Monitoring of glucose plays an essential role in the management of diabetes. However, to fully understand and meaningfully interpret glucose levels, additional information on context is necessary. Important contextual factors include data on behaviours such as eating, exercise, medication-taking and sleep, as well as data on mental health aspects such as stress, affect, diabetes distress and depressive symptoms. This narrative review provides an overview of the current state and future directions of precision monitoring in diabetes. Precision monitoring of glucose has made great progress over the last 5 years with the emergence of continuous glucose monitoring (CGM), automated analysis of new glucose variables and visualisation of CGM data via the ambulatory glucose profile. Interestingly, there has been little progress in the identification of subgroups of people with diabetes based on their glycaemic profile. The integration of behavioural and mental health data could enrich such identification of subgroups to stimulate precision medicine. There are a handful of studies that have used innovative methodology such as ecological momentary assessment to monitor behaviour and mental health in people’s everyday life. These studies indicate the importance of the interplay between behaviour, mental health and glucose. However, automated integration and intelligent interpretation of these data sources are currently not available. Automated integration of behaviour, mental health and glucose could lead to the identification of certain subgroups that, for example, show a strong association between mental health and glucose in contrast to subgroups that show independence of mental health and glucose. This could inform precision diagnostics and precision therapeutics. We identified just-in-time adaptive interventions as a potential means by which precision monitoring could lead to precision therapeutics. Just-in-time adaptive interventions consist of micro-interventions that are triggered in people’s everyday lives when a certain problem is identified using monitored behaviour, mental health and glucose variables. Thus, these micro-interventions are responsive to real-life circumstances and are adaptive to the specific needs of an individual with diabetes. We conclude that, with current developments in big data analysis, there is a huge potential for precision monitoring in diabetes.

**Graphical abstract:**

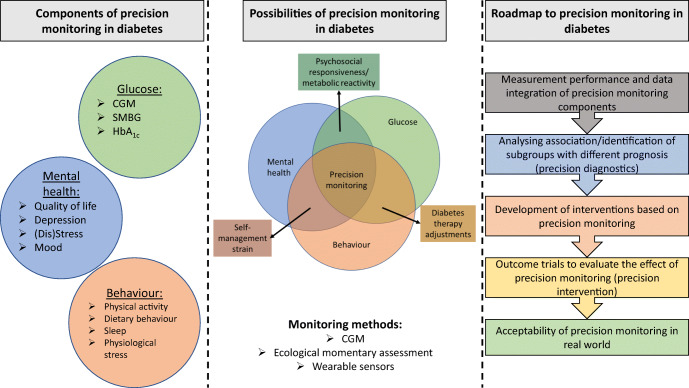

**Supplementary Information:**

The online version of this article (10.1007/s00125-022-05685-7) contains a slideset of the figures for download, which is available to authorised users.

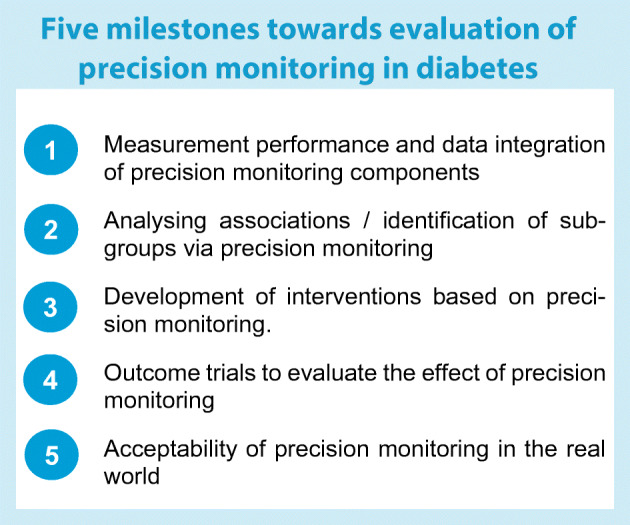



## Introduction

Diabetes is a condition for which monitoring is essential for diagnosis and therapy [1, 2]. Particularly with the introduction of insulin therapy a hundred years ago, it became increasingly important to monitor glucose levels to dose insulin accordingly [3]. However, it became apparent that attaining treatment targets and simultaneously avoiding hypoglycaemia requires the consideration of contextual factors such as eating and exercise. Historically, this was achieved by logbooks, in which glucose levels, insulin doses, food intake and exercise were recorded by hand [4]. In addition, factors such as sleep [5], mood [6], emotional stress [5], diabetes distress [7] and psychological comorbidities such as depression and eating disorders [7–9] proved to be relevant for diabetes outcomes.

Technological advances over the last decade made monitoring of blood glucose levels in the context of the above-mentioned factors easier. This can be seen by a rapid development of glucose sensors for continuous glucose monitoring (CGM) and the increasing availability of new devices, apps and additional sensors, which are able to monitor biological markers, self-management behaviour, sleep, eating patterns and psychological variables. In addition, the ability to integrate large datasets from these different sources and the intelligent processing and interpretation thereof is developing.

By combining CGM data with the monitoring of these contextual variables, precision monitoring in diabetes can be achieved. This is in line with the current ADA and EASD consensus statement that calls for precision monitoring. Based on the consensus statement, precision monitoring is defined as the multimodal assessment of glucose, behaviours, diet, sleep and psychophysiological stress [10].

In this narrative review, we provide an overview of precision monitoring in diabetes by monitoring of glycaemic control, self-care behaviour and mental health. Specifically, we want to address the following points: (1) how precision monitoring could be used to improve prediction of diabetes outcomes (precision prognostics); and (2) what intervention strategies can be derived by the insights gained from precision monitoring (precision therapeutics). Lastly, we discuss current gaps in knowledge, suggest future directions for precision medicine in diabetes, and present a possible roadmap to precision monitoring in diabetes.

## Precision monitoring in diabetes

Currently, there are three different methods for monitoring glucose: laboratory testing of blood glucose or HbA_1c_; self-monitoring of blood glucose (SMBG); and CGM.

Laboratory tests are the most precise measurement of blood glucose; they are usually performed for diagnostic or monitoring purposes and for adjustment or advancements of medical treatments.

SMBG became available in the 1970s and enabled people with diabetes to perform an intensified insulin therapy by autonomously adjusting insulin doses to their lifestyle (e.g. flexibility regarding time and size of their meals and exercise), leading to improved glycaemic control and better health outcomes [11]. Compared with laboratory tests, SMBG allows assessment of distribution and variation of glucose in addition to the level. However, the number of daily SMBG episodes, testing intervals and timings are decisive factors determining the potential of SMBG to identify glucose patterns.

These limitations of SMBG can be overcome by CGM, which provides interstitial glucose values every 5–15 min. This leads to a more complete picture of glucose control, especially during the night and after meals. The percentage of glucose values in the hypoglycaemic and hyperglycaemic ranges, as well as within a normal range, and glucose variability can be validly assessed. A consensus statement recommends certain thresholds for low and high glucose values as well as the percentage of values in the normoglycaemic range [12]. The beneficial effects of monitoring personal glucose levels are highlighted by the emergence and widespread adoption of CGM [13–15]. The in-depth glycaemic insights and direct feedback provided by CGM systems have proven efficacious in lowering elevated HbA_1c_ values and reducing the incidence of biochemical and clinical hypoglycaemia [16–18]. Further, research using CGM can either adopt a blinded protocol, in which the participant has no access to the recorded values, or open CGM, in which the participant can view and respond to glucose levels.

Kovatchev et al developed the low blood glucose index (LBGI), which can be used for SMBG and CGM data, a logarithmically weighted measure of low glucose values [19]. By combining the LBGI with clinical data on severe hypoglycaemic episodes, subgroups of people with type 1 diabetes and different risk levels for future hypoglycaemia were identified [20]. This might be a first contribution to precision prognostics, allowing the response of avoidance of low glucose values in subgroups with elevated LBGI. To simplify interpretation of SMBG data, Mazze et al developed the Ambulatory Glucose Profile (AGP) [21]. The AGP is able to create individually meaningful displays of glucose levels over the day, to provide information about the individual course of glucose during the day and night. However, clinically relevant subgroups of people with diabetes, or specific therapeutic interventions, were not identified using the AGP because of the lack of important context variables, such as timing of meals, exercise and medication intake/insulin injection.

Diabetes therapies and prognosis are highly dependent on self-management [22, 23]. To achieve near-normal glucose values, people with diabetes are required to keep track of much more than just glucose levels (e.g. administering insulin and/or taking medications, eating habits, exercise, foot care and maintaining general healthy lifestyle habits) [23]. These never-ending requirements of diabetes therapy and constant self-management can also be perceived as a burden and negatively affect mental health [9]. Subsequently, mental health problems, such as elevated distress due to diabetes [7, 24, 25], depressive symptoms [7, 26, 27] and overall reduced quality of life, are common in diabetes [28]. Quality of life is an important endpoint of diabetes, besides morbidity and mortality. Impaired mental health can also negatively affect self-care behaviours and quality of life, which all can be related to unfavourable prognosis [7, 29–32]. The important impact of mental health is also reflected in the most recent consensus statement of the EASD and ADA [33]. Thus, maintaining an optimal quality of life and preventing acute as well as long-term complications of diabetes require not only monitoring of glucose but also the simultaneous assessment and integration of contextual factors such as mental health, self-care behaviour and sleep. Questionnaires have long been the standard in assessing and monitoring mental health or behaviours. However, questionnaires have considerable limitations that can bias the whole assessment period; these include recall bias (e.g. trying to remember the last 2 weeks) and salient effects when a specific symptom, mood or emotion were particularly relevant [34]. Furthermore, ongoing precision monitoring is not easily possible with questionnaires since the automated integration of different data sources is currently not possible. These limitations can be overcome by ecological momentary assessment (EMA), a methodology that allows the repeated daily sampling of participants’ experiences and behaviours in their everyday lives [35]. EMA allows monitoring of mental health states and self-care behaviours as an important context of continuously measured glucose values, as current mood, affect, sleep quality and diabetes distress as well as other behaviours can be simultaneously assessed on a daily level and multiple times daily. Table 1 provides an overview of current monitoring methods and considerations for a precision monitoring approach.

**Table 1:** Monitoring in diabetes and considerations for precision monitoring

Monitoring method	Monitored variable	Mode of monitoring	Considerations for precision monitoring
Laboratory analysis	HbA_1c_ Glucose Lipids Markers of inflammation Genetic information	Active	Monitoring of risk-factors for complications
Self-monitored blood glucose	Current glucose level Distribution of glucose values	Active	Dose adaptation Definition of risk groups for acute complications Frequency of required measurements is uncertain
Blinded CGM	Retrospective daily glucose control Distribution of glucose values	Passive	Definition of risk groups for acute complications Glucose patterns Meeting of treatment targets No reactive measurement of glycaemic control Possibility of intermittent use is uncertain
Real-time CGM	Past glucose course Current glucose level Trend in glucose level Distribution of glucose values	Passive/active	Definition of risk groups for acute complications Glucose patterns Meeting of treatment targets Possibility of intermittent use is uncertain
EMA	Mental health (patient-reported outcomes): Stress Mood/Affect Diabetes distress Quality of life Depressive symptoms Diabetes symptoms Fear of hypoglycaemia	Active	Identification of impaired mental health Automated analysis resulting in meaningful variables needed Automated integration with glucose data needed Timing and duration of prompts is uncertain Number of daily prompts is uncertain Use of validated questions from established questionnaires is uncertain
	Self-care behaviour (self-report), eating: Meal size Timing Food choices Portion size Context of eating (e.g. stress eating, boredom) Disordered eating	Active	Effect of lifestyle interventions Motivation for lifestyle interventions Visibility of the effects of different foods on glucose Potential bias by socially desirable responses
	Self-care behaviour (self-report), treatment adherence: Timing of medication (e.g. insulin) No. of medications taken/injections Dosage of medication	Active	Effect of monitoring on adherence Potential bias by socially desirable responses
	Self-care behaviour (self-report), sleep: Sleep-in and wake-up time Sleep quality	Active	Impact of sleep quality on glucose metabolism (vice versa) Mental health and sleep Potential bias by socially desirable responses
Wearable sensor-wristbands	Physical activity: Steps Distance covered Heart rate Intensity Oxygen saturation	Passive	Effect of lifestyle interventions Motivation for lifestyle interventions Visibility of the effects of physical activity on glucose Correspondence to self-report Validity of data is difficult to ascertain Additional device(s) to wear
	Sleep: Sleeping hours Time in non-REM/REM Number of awakenings Breathing	Passive	Identification of sleep problems Objective variables in addition to perceived sleep quality Validity of data is difficult to ascertain Additional device(s) to wear
	Physiological arousal: Heart rate Heart rate variability Heart rhythm	Passive	Objective variables of stress responsiveness Validity of data is difficult to ascertain Additional device(s) to wear
Smart pens, pump data storage, electronic medication caps	Treatment adherence: Timing of medication (e.g. insulin) No. of medications taken/injections Dosage of medication	Passive	Detailed analysis of diabetes management Correspondence to self-report Costs Availability

In the following section, studies that investigate the associations between mental health, self-care behaviour, sleep and glycaemic control are described. An overview of the reviewed studies, including key study and methodological characteristics, is provided in Table 2. All, but one of the studies were observational, sharing the limitations of observational studies. Most studies used open CGM or SMBG.

**Table 2:** Overview of studies involving monitoring of mental health, behaviour and glycaemic control

Study	Monitoring method	Sample	Key outcomes	Methodological characteristics
Mood and glycaemic control				
Cox et al, 2007 [36]	SMBG	60 people with T1D	Postprandial excursions were associated with negative mood state and cognitive impairment	Observational Randomised Open label SMBG
Hermanns et al, 2007 [6]	CGM-blind	36 people with T1D	Higher glucose values were associated with less positive and more negative mood states Glycaemic variability showed no association with mood state	Observational Blinded CGM Multilevel analysis
Wagner et al, 2017 [37]	CGM-blind	50 people with T2D	Glycaemic variability had no association with mood state High and low glucose values were associated with negative affect	Observational Blinded CGM- Multilevel analysis
Skalf et al, 2009 [38]	SMBG	204 people with T2D	Negative mood predicted high fasting glucose the next day	Observational Open CGM Multilevel CGM
Shapira et al, 2021 [39]	SMBG	32 children / adolescents with T1D	Positive affect was associated with higher TIR, less time below range and less GV	Observational SMBG Multilevel analysis
Polonsky and Fortman, 2020 [40]	Open CGM	2019 people with T1D	Higher daily TIR was associated with better mood rating in the evening No association found between mood and GV	Observational Open CGM Multilevel analysis
Behaviour and glycaemic control				
Wagner et al, 2017 [37]	Blind CGM EMA	50 people with T2D	Higher variability in self-care was associated with more hyper- and hypoglycaemic values	Observational Blinded CGM Multilevel analysis
Moscovich 2019, [47]	EMA	83 adults with T1D	Negative affect prior to meal was associated with more binge eating Binge eating was associated with higher postprandial glucose values	Observational Open CGM Multilevel analysis
Cecilia-Costa et al, 2021 [67]	Questionnaire	169 children / adolescents with T1D	Negative affect and higher diabetes distress were associated with more binge-eating episodes Disordered executive function was associated with more disordered eating behaviour	Observational SMBG or CGM
Yang et al, 2020 [49]	mHealth devices	60 people with T2D	Three phenotypes: low, medium and high engagement Low engagement was associated with higher HbA_1c_	Observational SMBG 6 month follow-up
Sleep and glycaemic control				
Reutrakul et al, 2013 [68]	Sleep questionnaires	194 people with T2D	Lower sleep depth (<6 h) and unfavourable sleep chronotype were associated with higher HbA_1c_	Meta-analysis of observational studies Great heterogeneity
Knutson et al, 2011 [54]	Wrist actigraphy	40 people with T2D	Sleep fragmentation was associated with higher fasting glucose and higher HOMA index	Observational SMBG Multicentric

GV, glucose variability; T1D, type 1 diabetes; T2D, type 2 diabetes; TIR (time-in-range; glucose level 3.9–10 mmol/l)

### Mental health and glycaemic control

A study by Cox et al demonstrated postprandial effects of glucose on mood, with the rate of rise in postprandial glucose excursions being directly associated with negative mood (depressive and anxiety symptoms) but not with positive mood [36]. An early study by Hermanns et al, using blinded CGM and multiple mood assessment over 2 days, demonstrated that glucose levels collected 60 min prior to mood ratings showed significant negative associations with hedonic tone (i.e. happy) as well as positive associations with energetic arousal (i.e. active) and tension (i.e. anxious) [6]. Interestingly, this study did not find significant associations between glycaemic variability and mood. This matches with recent findings by Wagner et al in which glycaemic variability, assessed by blinded CGM over 7 days, showed no associations with positive nor negative affect [37]. Overall, Wagner et al only found associations between glycaemic variables (e.g. mean glucose, per cent hypo- or hyperglycaemic values) and negative affect, but not positive affect [37]. These findings indicate that higher glucose levels and negative mood appear to show a stronger association than glucose and positive mood. Support for this comes from Skaff et al, who demonstrated that, in people with type 2 diabetes, negative mood on one day was predictive of higher fasting glucose on the next day, while positive mood had no effect [38].

In teenagers with type 1 diabetes, however, a recent study by Shapira et al found that positive affect was associated with a higher frequency of in-range blood glucose levels and lower likelihood of hypoglycaemic values, as well as less glucose fluctuation. It should be noted that these findings were specific for those teenagers with HbA_1c_ ≤8% (63.9 mmol/mol) [39]. In adults with type 1 diabetes, Polonsky and Fortman demonstrated that over a period of 2 weeks, increases in daily time-in-range were significantly associated with better mood ratings in the evening [40]. However, mood ratings were not associated with daily changes in time-in-hypoglycaemia or with glycaemic variability.

A recent systematic review also concluded that associations between mood and glucose variability could not be convincingly shown. However, this review indicated a significant direct relationship between postprandial glucose and negative mood in people with type 2 diabetes and a potential positive effect of lower glucose variability on depressive mood in adults with type 1 diabetes [41].

In summary, there seems to be growing evidence that negative mood states are associated with elevated or low glucose values, while glucose values within a normal range are rather coincident with positive mood states and less negative affect. The causality and directionality of these relationships require additional study (e.g. by the use of vector autoregression methods [42, 43]). Further analysis of the relationship between mental health aspects and glucose levels, as well as excursions, is needed [44]. Nice examples of such analyses are current studies combing EMA and CGM: the FEEL-T1D study in the USA [45]; the DIA-LINK studies in Germany [44]; and the international Hypo-METRICS study within the Hypo-RESOLVE Research Consortium [46]. Such work could support the identification of subgroups of individuals whose mental health might be more strongly influenced by the course of glucose compared with others whose glycaemic control might not be strongly associated with mental health. From the point of view of ‘precision therapeutics’, different therapy strategies might be offered to the two subgroups: for the first subgroup interventions to improve mental health could be more effective when including the diabetes context; and for the latter subgroup improvement of glycaemic control and mental health could be addressed independently.

### Behaviour and glycaemic control

EMA has also been used to study the effects of behaviour on glycaemic control. Wagner et al used blinded CGM for 7 days in Latinos with type 2 diabetes and showed that higher variability in self-care behaviour was associated with a higher percentage of glucose values out of range (either <3.9 mmol/l or >10 mmol/l, particularly values in the hyperglycaemic range (>180 mmol/l) [37]. The consequence was an association between greater self-care behaviour and less glucose variability. This is corroborated by Shapira et al, who demonstrated that stronger negative affect was associated with less optimal self-care (fewer blood glucose checks), especially for teenagers with HbA_1c_ levels >8% (63.9 mmol/mol) [39].

In a study analysing binge eating in type 1 diabetes, people with higher negative affect, guilt, frustration or diabetes distress had a higher risk for binge-eating episodes. This, in turn, led to higher postprandial glucose excursions [47]. In a further step, it might be worth examining whether addressing these negative emotions in people prone to binge eating could help to prevent binge-eating episodes, thereby positively influencing glycaemic control. Another variable playing a potential role is that of executive function problems, which have been found to be associated with disordered eating behaviour [48].

An interesting study reports on digitally phenotyping the monitoring of self-care behaviour in people with type 2 diabetes [49]. Based on participants’ engagement with multiple mHealth devices used for monitoring, the authors found three distinct digital phenotypes: 33% had low and waning engagement, while the rest had either medium engagement or consistently high engagement. The authors found that being in the low and waning engagement group was associated with younger age, female sex, non-white race, lower income and higher baseline HbA_1c_ [49].

The automated assessment and integration of key self-care behaviours such as medication intake and administration of injections, and physical activity and eating, could facilitate consideration of these key behaviours in diabetes therapy. Monitoring of behaviours shows that there are direct links between self-care and glycaemic control [37, 47]. Identification of people with problems in self-care behaviours might enhance precision therapeutics since these people can then be offered specific support. Identification of subgroups of people who show a strong association between mental health problems and problematic self-care behaviours may allow for early intervention before deficits of self-care become obvious and damaging. However, it must be considered that subtyping of groups solely based on self-care behaviour might be difficult due to high variability of behaviour, often dependent upon changing life circumstances.

### Sleep and glycaemic control

Sleep is one of the most influential factors in hormonal regulation and circadian rhythm. Unsurprisingly, sleep disturbances are associated with a wide range of physiological and psychological problems (e.g. diabetes, depression). Notably, diabetes self-management efforts using modern devices can also disrupt sleep with, for instance, CGM glucose alerts or pump alarms. In addition, nocturnal hypoglycaemia can also negatively affect sleep quality [50]. Observational data suggest that sleep disturbances or impaired sleep quality are more prevalent in people with diabetes [51]. Indeed, there is evidence that reduced sleep is related to the occurrence of type 2 diabetes, heightened inflammation, insulin resistance and appetite and weight gain [52–54]. Furthermore, insomnia and obstructive sleep apnoea are more prevalent in people with vs without diabetes (prevalence estimates of about 50% in those with diabetes) [55, 56]. Meta-analyses suggest that shorter sleep duration and lower sleep quality in particular are associated with less-than-optimal glycaemic control [56, 57]. In adults with type 1 diabetes, those who had poor self-reported sleep quality or who slept for ≤6 h had higher HbA_1c_ [56]. In a meta-analysis of adults with diabetes, a U-shaped association was found, with shorter sleep duration (<6 h) and longer sleep duration (>8–9 h) being associated with higher HbA_1c_ [57].

Wearables can be used to assess sleep behaviour and are especially useful for assessing sleep duration and number of awakenings, as well as duration of rapid-eye-movement (REM) and non-REM sleep [58]. Thus, monitoring of sleep patterns could also inform therapy adjustments by identifying people with disordered sleep.

In general, the literature shows that sleep is an important determinant of the course of diabetes. Sleep quality is associated with less-than-optimal glycaemic control. Subtyping of people with diabetes according to their sleep characteristics might enhance precision prognostics. Monitoring of sleep in the context of glycaemic control as well as mental health might also indicate therapeutic starting points.

## Precision prognostics and diagnosis

Monitoring of glucose levels, (self-care) behaviours and mental health can provide a near-complete picture of glucose patterns, corresponding behaviours and the subjective mental states of people with diabetes at a certain point in time. Combining and synthesising these three data sources could contribute to a better prediction of long-term diabetes outcomes such as morbidity, mortality and quality of life.

**Fig. 1:**
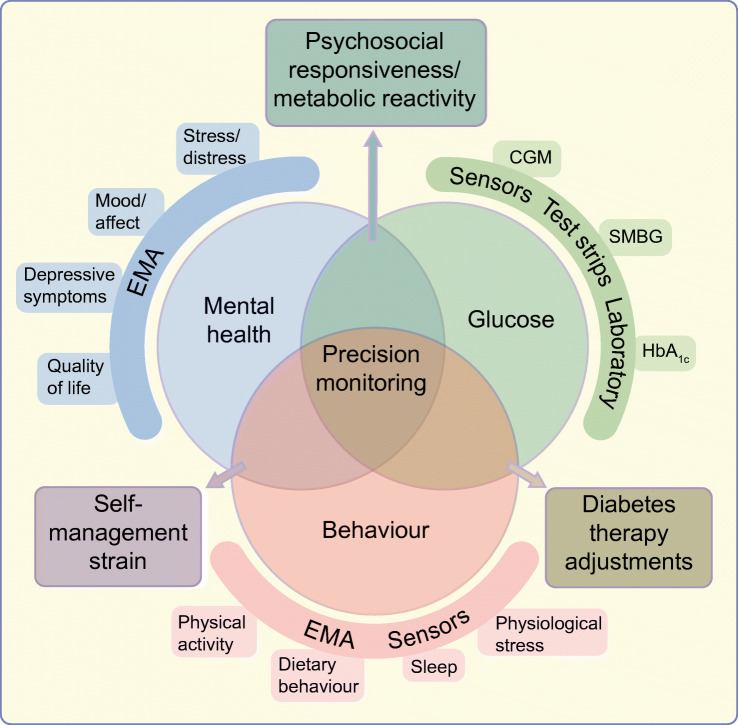
Conceptual model of multidimensional monitoring in diabetes (glucose, self-care behaviour, mental health). Methods of monitoring glycaemic variables (green), behaviour and physiological variables (orange) and psychological variables (blue) are shown. Combination of two or all of these monitoring areas can contribute to precision monitoring in diabetes, as shown by overlap of the circles. Rectangular boxes show precision monitoring areas (associations among glucose control, mental health and behaviour, which can affect psychosocial and metabolic responsiveness, lead to self-management strain and provide opportunities for diabetes therapy adjustments). This figure is available as part of a downloadable slideset

**Fig. 2:**
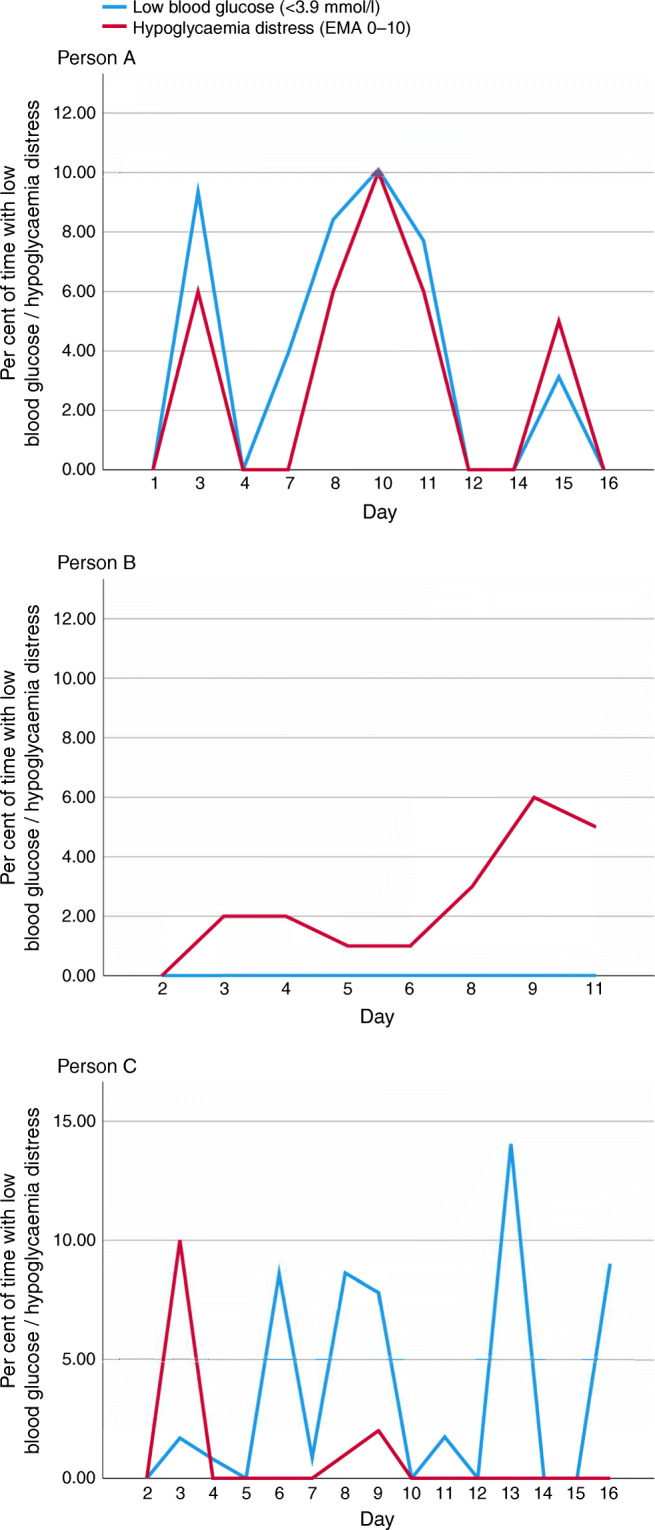
The association between perceived hypoglycaemia distress and exposure to low glucose values. Data arising from three different case studies are shown. Low blood glucose, assessed by CGM, was defined as <3.9 mmol/l; perceived hypoglycaemia distress was assessed by EMA 0–10. Data are taken from three individual participants in the DIA-LINK study led by NH, DE, ASc and BK (unpublished). This figure is available as part of a downloadable slideset

Figure 1 offers a conceptual representation of different aspects of precision monitoring in diabetes. Combination of all three or two of the monitoring areas may encompass precision monitoring [10]. Of course, the approach taken to precision monitoring should be tailored to the individual and may not be entirely perfect for each person with diabetes nor suitable for all subgroups. For people without indication of impaired mental health, monitoring of behaviour and glucose could lead to therapy adjustments regarding medication or behavioural changes. For people with a strong association between mental health variables, such as diabetes distress, and glycaemic outcomes, a different prognosis regarding the future course of glycaemic control or intervention might result when compared with people with a weak or no association between glucose and diabetes distress. This is exemplified by the simultaneous monitoring of hypoglycaemia-related distress and exposure to low glucose values. Figure 2 depicts data from three people with different associations between perceived hypoglycaemia distress (assessed via EMA) and exposure to low glucose values (assessed by CGM). In the individual shown in Fig. 2a, the pattern of perceived subjective distress closely mirrors that of low glucose levels. A promising strategy for reducing perceived distress might be to avoid low glucose values by medical interventions such as adjustment of glycaemic targets, change of medication dose, the use of insulin pump therapy or automated insulin delivery (AID) systems, or diabetes education. The individual whose data are shown in Fig. 2b might not profit from these medical interventions regarding hypoglycaemia distress, since subjective distress seems unrelated to the actual exposure to low glucose values. Here, the identification of different triggers of hypoglycaemic distress (e.g. thoughts, high level of general anxiety) might be more appropriate. Figure 2c shows data from an individual who has high exposure to low glucose values but no subjective hypoglycaemia distress. This person might benefit from a different risk appraisal regarding hypoglycaemia. These examples demonstrate how precision monitoring can inform precision diagnostics and may help to suggest differential therapeutical targets and starting points.

Thus, the longitudinal monitoring of variables from key areas (behaviour, glycaemic control and mental health) allows the aggregation of these variables at a within-person level and the statistical analysis of individual courses. Assessment of such idiosyncratic associations between variables of the three key areas may also enhance precision diagnostics and prognostics in diabetes at an individual level. An individual longitudinal neural network analysis with multiple assessments over time or latent class growth analysis [59] may provide such idiosyncratic courses over time. At a between-person level, the magnitude of the idiosyncratic associations can also be combined with genetic, metabolic or other biological–medical factors by cluster analytical methods. This would allow subtyping of people with diabetes based on precision monitoring and provide individual targets for interventions.

## Precision therapeutics in diabetes

The identification of ‘glycaemic–behavioural–mental health patterns’ via precision monitoring can lead to different treatment approaches and better outcomes as described above. An intriguing possibility is the automated integration of monitoring results into treatment decisions, a concrete example being the use of AID systems in type 1 diabetes. Studies showed that AID systems could increase time-in-range (glucose levels of 3.9–10 mmol/l) by approximately ten percentage points [60–62]. However, in these studies time-in-range usually hovered around 70% [60–62], possibly indicating that the full potential of AID control is not yet achieved. One problem might be that relevant contextual factors such as eating behaviour, exercise and experience of stress are not sufficiently considered in the current systems. The automated integration of monitoring results regarding stress, exercise and amount of carbohydrates might help to inform the algorithms of AID systems about upcoming glucose excursions, allowing earlier response. Integration of this contextual information might also help to improve outcomes in people with type 2 or type 1 diabetes who are using less-complex therapies (e.g. multiple daily insulin injections) or other decision support systems (e.g. smart pens, bolus calculators).

Precision monitoring of glucose, behaviour and mental health could also be used to trigger interventions automatically. One possible approach involves so-called just-in-time adaptive interventions [63]. This comprises micro-interventions that are automatically triggered when a specific problem is identified by precision monitoring. Thus, just-in-time adaptive interventions could be tailored to a specific person by being adaptive to the current circumstances (e.g. high glucose, low adherence, high psychological stress) and responsive to the moment a problem is identified (e.g. suggestion of a micro-intervention on a person’s smartphone). These micro-interventions could not only target glycaemic control but also self-care behaviour and mental health.

In severe mental illness with a cyclic course (e.g. bipolar depression, psychosis), which often presents insurmountable hurdles to optimal glycaemic control, precision monitoring of symptoms or other variables such as activity, mobility or communication behaviour could support early detection of a relapse. This would inform ‘in-time’ (pharmacological) treatment intensification or psychological interventions such as cognitive behaviour therapy or mindfulness-based cognitive therapies, which have proven to be effective in treating severe mental illness and preventing deterioration of glycaemic control [64, 65].

## Roadmap to precision monitoring

There are several knowledge gaps that need to be addressed in order to achieve precision monitoring in diabetes:

1. More information is needed regarding the measurement performance, accuracy and reliability of sensor data yielded by monitoring of glucose, behaviour and mental health variables. Using EMA in routine care also raises the question of the clinical validity of EMA results compared with classical psychometric measurements by questionnaire or interview.
2. The stability and directionality of associations between glucose, behaviour and mental health is as unresolved as the question of directionality. Another open question is the potential of clustering people based on individualised associations between these key monitoring areas into meaningful subgroups regarding prognosis and treatment. Further, more information is needed on whether all three key monitoring areas (behaviour, mental health, glucose outcomes) should be weighted equally or differentially.
3. Mechanisms of change that can be targeted by interventions need to be identified. The impact of therapeutic use of feedback regarding individual associations also needs to be addressed in pilot-studies and tailored algorithms to control such feedback needs to be developed. Avoidance of potential information overload and assessment burden must be taken into account when developing such interventions based on precision monitoring.
4. There is a need for rigorous testing of newly developed interventions in controlled studies (ideally in randomised controlled studies). Factorial and platform trials may provide a greater cost-effectiveness of such testing.
5. Medical, demographic and social variables (e.g. age, sex, diabetes type, socioeconomic status, comorbidities, cognitive decline) often interact with the readiness to monitor certain aspects of living with diabetes as well as with the readiness to take part in intervention studies. Real-world studies and healthcare research can provide data about the efficacy of the newly developed interventions under conditions of routine care and also about cost-effectiveness. Particularly, these studies can provide a clearer picture of which subgroups of people can profit from precision monitoring in diabetes.

**Fig. 3:**
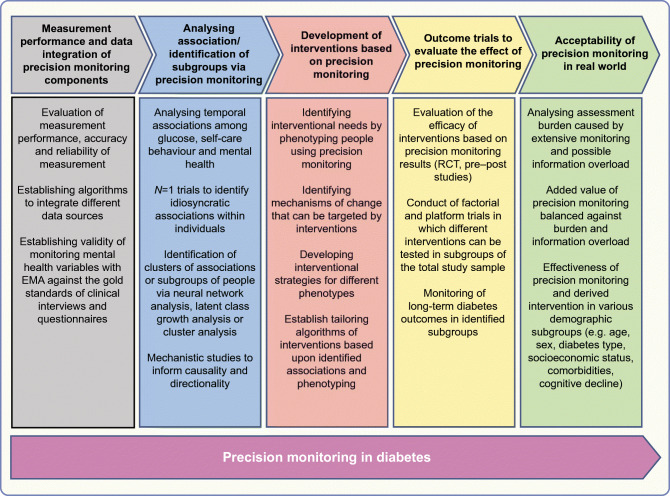
Roadmap suggesting studies necessary for achieving precision monitoring in diabetes. This figure is available as part of a downloadable slideset

Precision monitoring in diabetes is a new and developing field of research and clinical care. A possible roadmap towards precision monitoring, addressing the identified gaps, is depicted in Fig. 3. We suggest five evaluative milestones in the Text box, above. This roadmap may inform the way to precision diagnostics and precision therapeutics that are based on precision monitoring.

## Conclusion

Taken together, monitoring of self-care behaviour and mental health can significantly enrich glucose data by providing context to the glucose values. Interpreting glucose while considering self-care behaviours and mental health issues will become more precise and could facilitate clinically meaningful inferences and opportunities for therapy adjustments that match the specific needs of an individual. Use of precision monitoring could allow identification of psychobehavioural glucotypes, each of which could then benefit from a precision medicine approach to treatment. To achieve this, standards for monitoring and interpretation of self-care behaviours and mental health must be developed, based on the example of CGM [66]. Furthermore, there is need for the automated combination and integration of the data sources using technological as well as artificial intelligence solutions. While precision monitoring is not yet established, it is a next step towards giving people with diabetes and healthcare professionals the tools to better understand the intricacies of diabetes therapy and help inform appropriate management.

## Supplementary Information

ESM (PPTX 623 kb)

## Abbreviations

AGP: Ambulatory Glucose Profile
AID: Automated insulin delivery
CGM: Continuous glucose monitoring
EMA: Ecological momentary assessment
LBGI: Low blood glucose index
REM: Rapid-eye-movement
SMBG: Self-monitoring of blood glucose
