# Supplementary material for: Coordination of glucose monitoring, self-care behaviour and mental health: achieving precision monitoring in diabetes
Source: Diabetologia. 2022 Apr 5;65(11):1883–94. doi: 10.1007/s00125-022-05685-7 (PMC9522821; doi:10.1007/s00125-022-05685-7)
Supplement: Supplementary file 1 — (PPTX 623 kb) [file 125_2022_5685_MOESM1_ESM.pptx]

## Slide 1
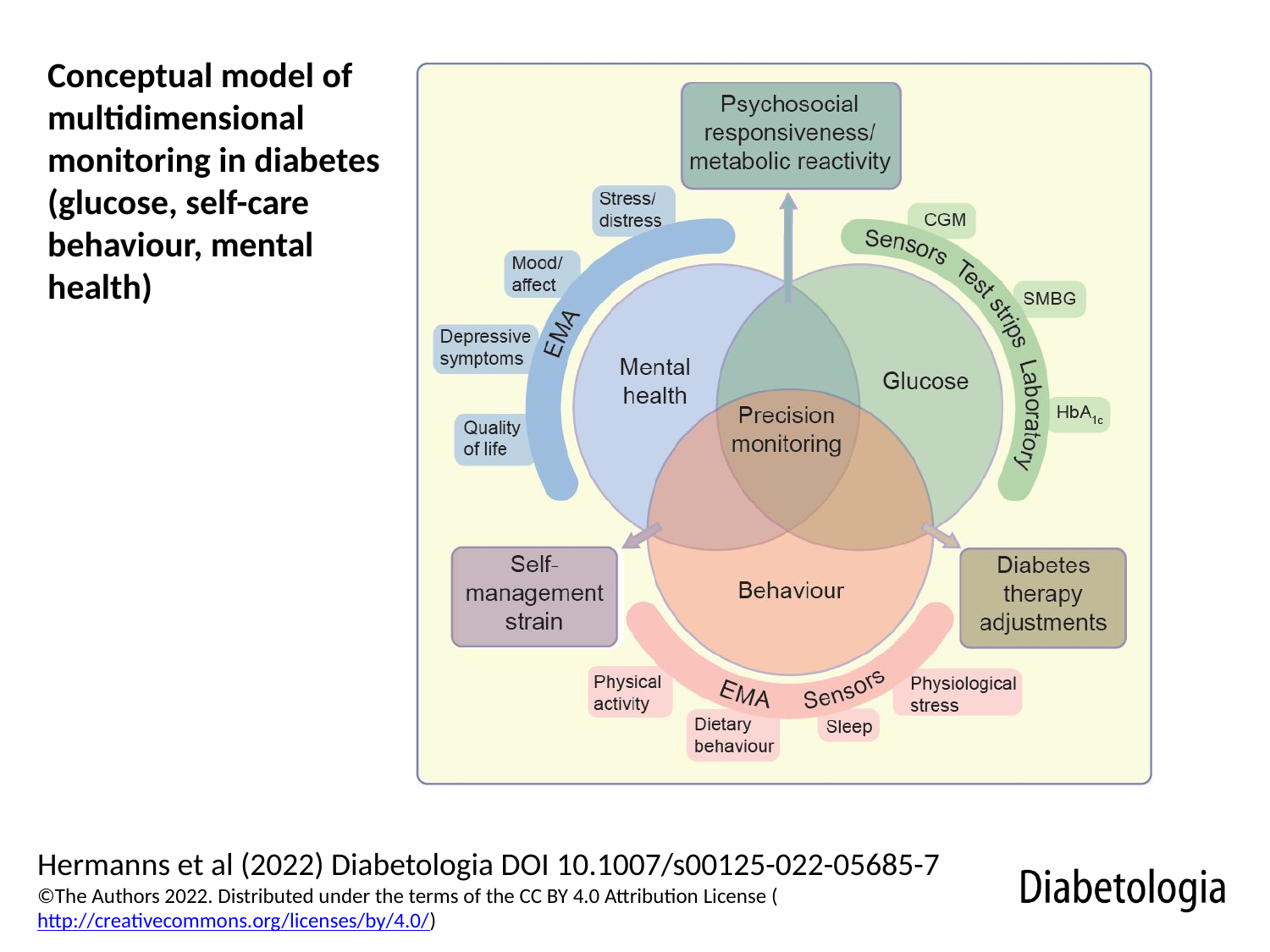

Conceptual model of multidimensional monitoring in diabetes (glucose, self-care behaviour, mental health)
Hermanns et al (2022) Diabetologia DOI 10.1007/s00125-022-05685-7
©The Authors 2022. Distributed under the terms of the CC BY 4.0 Attribution License (http://creativecommons.org/licenses/by/4.0/)

## Slide 2
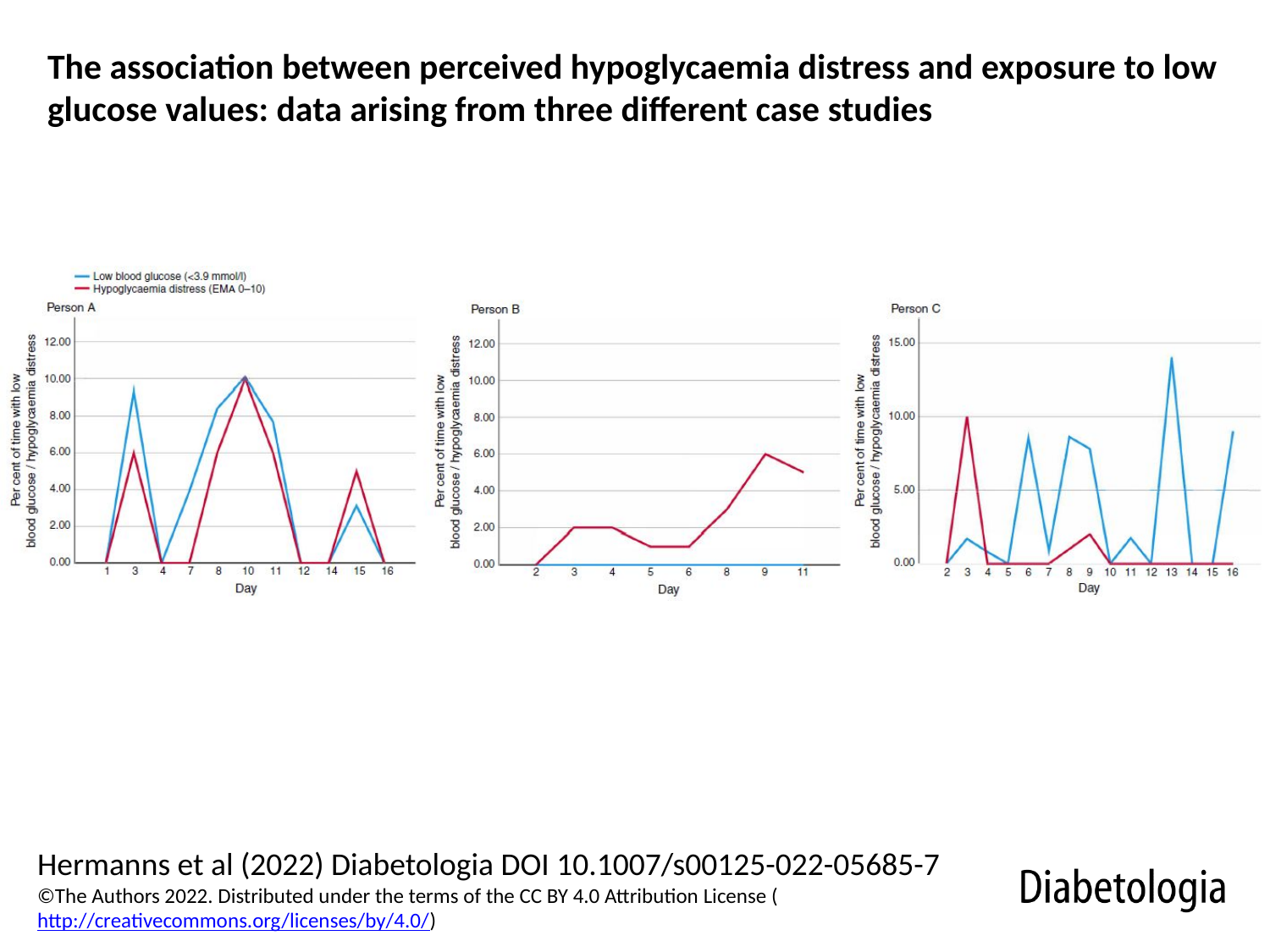

The association between perceived hypoglycaemia distress and exposure to low glucose values: data arising from three different case studies
Hermanns et al (2022) Diabetologia DOI 10.1007/s00125-022-05685-7
©The Authors 2022. Distributed under the terms of the CC BY 4.0 Attribution License (http://creativecommons.org/licenses/by/4.0/)

## Slide 3
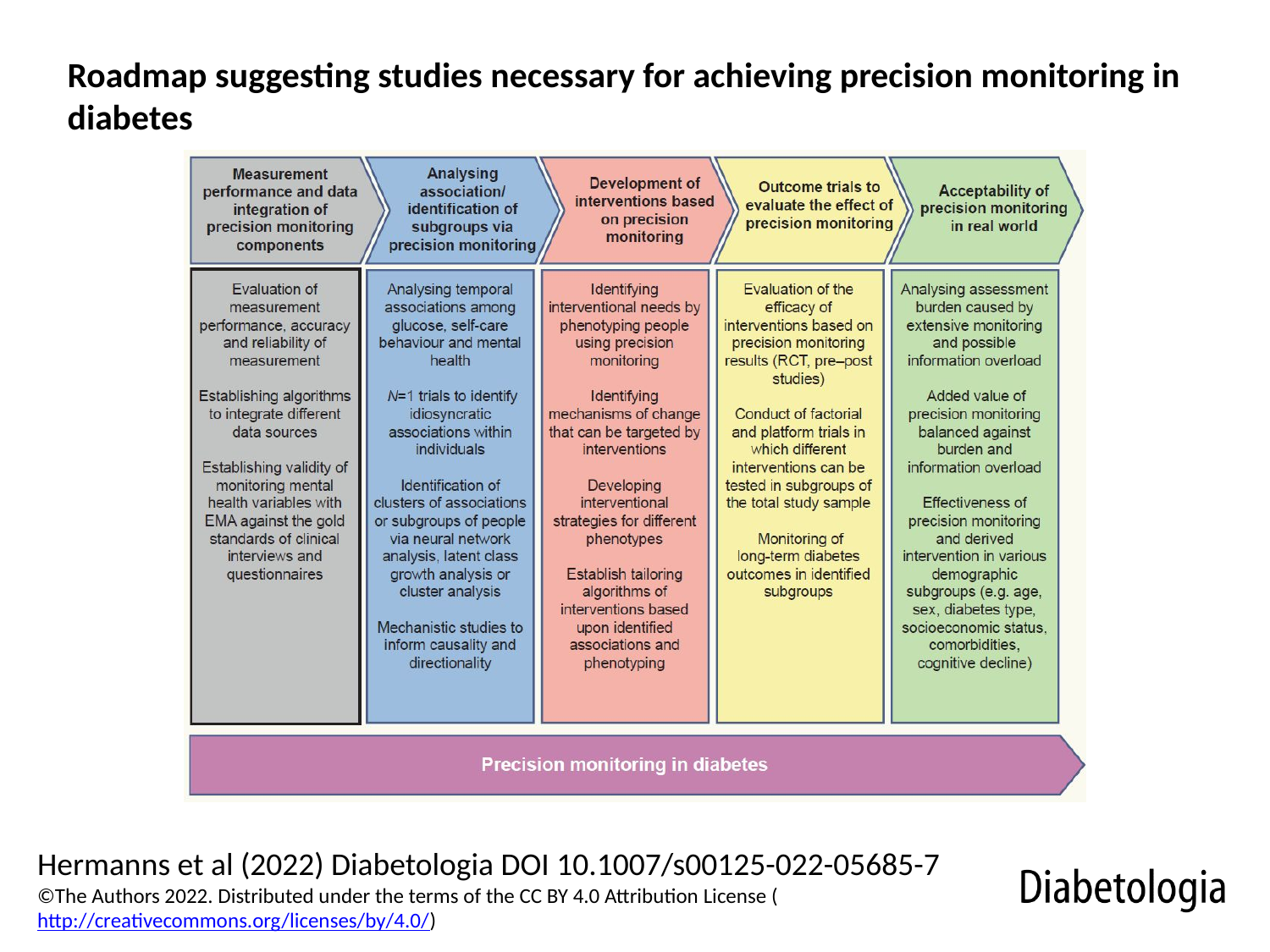

Roadmap suggesting studies necessary for achieving precision monitoring in diabetes
Hermanns et al (2022) Diabetologia DOI 10.1007/s00125-022-05685-7
©The Authors 2022. Distributed under the terms of the CC BY 4.0 Attribution License (http://creativecommons.org/licenses/by/4.0/)
